# New influenza A Virus Entry Inhibitors Derived from the Viral Fusion Peptides

**DOI:** 10.1371/journal.pone.0138426

**Published:** 2015-09-18

**Authors:** Wenjiao Wu, Dongguo Lin, Xintian Shen, Fangfang Li, Yuxin Fang, Kaiqun Li, Tianrong Xun, Guang Yang, Jie Yang, Shuwen Liu, Jian He

**Affiliations:** School of Pharmaceutical Sciences, Southern Medical University, 1838 Guangzhou Avenue North, Guangzhou, 510515, P. R. China; Shanghai Medical College, Fudan University, CHINA

## Abstract

Influenza A viral (IAV) fusion peptides are known for their important role in viral-cell fusion process and membrane destabilization potential which are compatible with those of antimicrobial peptides. Thus, by replacing the negatively or neutrally charged residues of FPs with positively charged lysines, we synthesized several potent antimicrobial peptides derived from the fusogenic peptides (FPs) of hemagglutinin glycoproteins (HAs) of IAV. The biological screening identified that in addition to the potent antibacterial activities, these positively charged fusion peptides (pFPs) effectively inhibited the replication of influenza A viruses including oseltamivir-resistant strain. By employing pseudovirus-based entry inhibition assays including H5N1 influenza A virus (IAV), and VSV-G, the mechanism study indicated that the antiviral activity may be associated with the interactions between the HA2 subunit and pFP, of which, the nascent pFP exerted a strong effect to interrupt the conformational changes of HA2, thereby blocking the entry of viruses into host cells. In addition to providing new peptide “entry blockers”, these data also demonstrate a useful strategy in designing potent antibacterial agents, as well as effective viral entry inhibitors. It would be meaningful in treatment of bacterial co-infection during influenza pandemic periods, as well as in our current war against those emerging pathogenic microorganisms such as IAV and HIV.

## Introduction

To date, influenza A viruses (IAV) are one of the critical human respiratory pathogens that not only cause morbidity and mortality seasonally, but also create serious public fear and economic loss worldwide. The recent pandemics and outbreaks of disease, such as H5N1 avian influenza (bird flu) [1] and H7N9 influenza A pandemics in China [2] have posed a significant impact on the international community, and presented an urgent need to develop new and effective anti-IAV agents. It has been well known that apart from the bactericidal activity, many antimicrobial peptides (AMPs) are also effective against pathogenic viruses thus making them potential therapeutics in the prophylaxis and treatment of viral infections [3]. So far, three modes of action of antimicrobial peptides have been proposed with respect to viruses: direct virolysis, inhibition of transcription from the long terminal repeat (LTR) promoter, and block of cell entry by binding to cell surface receptors [4]. This means that a novel antiviral agent could be generated from the structures of antimicrobial peptides. Started from these points and encouraged with the success of HIV peptide entry blocker of enfuvirtide (Fuzeon) [5], we reviewed the structural characteristics of some fusion peptides derived from hemaglutinin glycoproteins of influenza A viruses with the attempt to create novel antimicrobial agents against such emerging pathogens.

The fusion peptides of enveloped viruses such as HIV and influenza virus usually play an important role in viral entry into host cells. Influenza A fusion peptides (FPs) are the segments of hemagglution glycoproteins (HAs) and refer to peptides being able to destabilize the membrane bilayer and increase the surface tension [6]. The HA is one of the major surface glycoproteins of the influenza viruses and is responsible for the binding and fusion of the viral envelope with the infected cell’s membrane in the initial step of infectious process. Structurally, HA forms a homo-trimer with each of the monomers containing two subunits, HA1 and HA2, linked by a disulphide bond. The HA1 subunit is for the initial step of binding the virus with sialic acid on host cells, while the HA2 is for fusion [7]. For the HA2 subunit, the N-terminal segment consisting of 23 amino acids is the so-called fusion peptides (FP) [7].

As shown in Table 1, all FPs from IAV are hydrophobic and possess multiple glycine residues. Notably, the amino acid sequences of these peptides are extremely conserved among the 16 recognized subtypes, especially for HA2 residues 1–11 in the N-terminal region [8]. Their secondary structures are reported as amphipathic α-helical or partial α-helical when interacting with membrane, which is similar to that of conventional antimicrobial peptides, such as ADP1 [9–10]. However, in contrast to most AMPs, all FPs from HAs are negatively charged, as listed in Table 1. Considering the critical role of the net positive charge in the conventional AMPs, we then turned several negatively charged fusion peptides into “positively charged” pseudo-fusion peptides (pFPs) by replacing negatively or neutrally charged residues with lysines, while most other residues remained untouched.

**Table 1:** Fusion peptide sequences from influenza A virus strains*.

	1	2	3	4	5	6	7	8	9	10	11	12	13	14	15	16	17	18	19	20	21	22	23
**H1**	G	L	F	G	A	I	A	G	F	I	E	G	G	W	T	G	M	I	D	G	W	Y	G
**H2**	**-**	**-**	**-**	**-**	**-**	**-**	**-**	**-**	**-**	**-**	**-**	-	-	-	Q	-	-	V	-	-	-	-	-
**H3**	**-**	**-**	**-**	**-**	**-**	**-**	**-**	**-**	**-**	**-**	**-**	N	-	-	E	-	-	-	-	-	-	-	-
**H4**	**-**	**-**	**-**	**-**	**-**	**-**	**-**	**-**	**-**	**-**	**-**	N	-	-	Q	-	L	-	-	-	-	-	-
**H5**	**-**	**-**	**-**	**-**	**-**	**-**	**-**	**-**	**-**	**-**	**-**	-	-	-	Q	-	-	V	-	-	-	-	-
**H6**	**-**	**-**	**-**	**-**	**-**	**-**	**-**	**-**	**-**	**-**	**-**	-	-	-	-	-	-	-	-	-	-	-	-
**H7**	**-**	**-**	**-**	**-**	**-**	**-**	**-**	**-**	**-**	**-**	**-**	N	-	-	E	-	L	V	-	-	-	-	-
**H8**	**-**	**-**	**-**	**-**	**-**	**-**	**-**	**-**	**-**	**-**	**-**	-	-	-	S	-	-	-	-	-	-	-	-
**H9**	**-**	**-**	**-**	**-**	**-**	**-**	**-**	**-**	**-**	**-**	**-**	-	-	-	P	-	L	V	A	-	-	-	-
**H10**	**-**	**-**	**-**	**-**	**-**	**-**	**-**	**-**	**-**	**-**	**-**	N	-	-	E	-	-	V	-	-	-	-	-
**H11**	**-**	**-**	**-**	**-**	**-**	**-**	**-**	**-**	**-**	**-**	**-**	-	-	-	P	-	L	-	N	-	-	-	-
**H12**	**-**	**-**	**-**	**-**	**-**	**-**	**-**	**-**	**-**	**-**	**-**	-	-	-	P	-	L	V	A	-	-	-	-
**H13**	**-**	**-**	**-**	**-**	**-**	**-**	**-**	**-**	**-**	**-**	**-**	-	-	-	P	-	L	-	N	-	-	-	-
**H14**	**-**	**-**	**-**	**-**	**-**	**-**	**-**	**-**	**-**	**-**	**-**	N	-	-	Q	-	L	-	-	-	-	-	-
**H15**	**-**	**-**	**-**	**-**	**-**	**-**	**-**	**-**	**-**	**-**	**-**	N	-	-	E	-	L	-	-	-	-	-	-
**H16**	**-**	**-**	**-**	**-**	**-**	**-**	**-**	**-**	**-**	**-**	**-**	-	-	-	P	-	L	-	N	-	-	-	-

* The H1 subtype is used as the reference sequence for this figure, and dashes for other fusion peptide sequences indicate direct homology to the H1 sequence, and changes from the H1 reference sequence are noted with the single letter amino acid code

As reported in our previous work [11], the newly generated positively charged fusion peptides (pFPs) exhibited potent antimicrobial activities against a broad variety of bacteria including gram positive and gram negative bacteria. In addition, the CD spectral analyses and computer simulation showed that both the indigenous fusion peptide (FP, negatively charged) and the newly created peptides (pFP, positively charged) had a similar secondary structure, and the change in net charge didn’t induce significant change in the secondary structure [12].

Given a highly conserved region in HA, FPs play a critical role in mediating virus-cell fusion events, thus the local region of fusogenic peptide on HA2 would be a potential target for antiviral intervention [13]. Based on this deduction, we then evaluated their antiviral activities by testing the influenza A viral strains of *A/Puerto Rico/8/34* (H1N1) and *A/Aichi/2/68* (H3N2), meanwhile, we also investigated the mechanism of action by employing various pseudoviruses based assays. The results showed that these peptides might be able to specifically block the entry of influenza A viruses into host cells, thus providing promising lead molecules to develop as potential antiviral agents. In this paper, we report on the anti-IAV activity and the possible mechanism of these peptides.

## Materials and Methods

### Organisms

Madin Darby Canine Kidney (MDCK) cells and 293T cells were obtained from the American Type Culture Collection (ATCC) and cells were grown in Dulbecco’s Modified Eagle Medium (DMEM) containing glutamine, supplemented with 10% fetal bovine serum (FBS). The influenza *A/Puerto Rico/8/34* (H1N1) and *A/Aichi/2/68* (H3N2) viruses were propagated in the allantoic cavities of 9-day-old embryonated hen eggs at 37°C. After harvest, the virus titer was determined through the analysis of the 50% tissue culture infective dose (TCID_50_) on MDCK cells and evaluated using the method developed by Reed and Muench [14].

### Peptide synthesis

Peptides were synthesized on an ABI 433A peptide synthesizer with 0.1 mmol scale by using standard Fmoc solid phase protocols on Rink Amide MHBA resin as described previously [15]. The molecular weight of each peptide was confirmed by electrospray ionization mass spectrometry (ESI-MS, Waters, USA), and the purity of peptides was analyzed with Shimadazu 10A HPLC instrument on a C18 column (250 × 4.6 mm, Shimadzu, Japan), with the purity *ca*. 90%.

### Viral cytopathic effect (CPE) inhibition assay

Antiviral activities of peptides were evaluated by the viral cytopathic effect inhibition assay as previously reported [16]. Briefly, influenza *A/Puerto Rico/8/34* (H1N1) and *A/Aichi/2/68* (H3N2) viruses at 100 TCID_50_ were mixed with peptides at indicated concentration and incubated at 37°C for 30 min respectively, and then the virus-peptide mixtures were added to the MDCK cells and incubated for another 60 min. After incubation, the supernatants were discarded and the cells were washed twice with PBS (phosphate buffer saline) to remove unabsorbed virus followed by adding virus growth medium (serum free DMEM supplemented with 1 μg/mL TPCK-trypsin). At 48 h post-infection, cell viability was measured with MTT ((3-(4,5-dimethyl-thiazol-2-yl)-2,5-diphenyltetrazolium bromide) assay.

To measure cell viability, 100 μL of MTT (0.5 mg/mL, diluted in culture medium) was added into each well and incubated at 37°C for 4 h. Reduced MTT (formazan) was extracted with acidic isopropanol. Absorbance at wavelengths of 570 nm was read on a microtiter plate reader (Genios Pro Tecan, Swiss). After subtraction of values of background, dose response curves of half-log concentration vs. percentage of protection were generated, from which half maximal inhibition concentration (IC_50_) was calculated.

### Quantitative real-time PCR assay

The inhibition of viral HA gene replication was detected by quantitative real-time PCR with reported protocols [17]. As mentioned above, influenza *A/Puerto Rico/8/34* (H1N1) virus at 100 TCID_50_ was mixed with peptides at indicated concentration and incubated at 37°C for 30 min, then the virus-peptide mixtures were added to the confluent monolayers MDCK cells in 6-well plates and incubated for another 30 min. After incubation, the supernatants were discarded and the cells were washed twice with PBS followed by addition of virus growth medium (serum free DMEM supplemented with 1 μg/mL TPCK-trypsin and 0.2% BSA). At 24 h post-infection, the total RNA was extracted with TRIzol reagent, and quantified by UV spectrophotometer (Merinton SMA1000, USA). Then the total RNA was reverse transcribed into cDNA using PrimeScript RT reagent kit. Real-time PCR was performed in an ABI7500 real-time PCR instrument (Applied Biosystems, USA) with the SYBR Premix Ex Taq as the manufacturer’s instruction. HA expression was normalized to GAPDH gene, which stably expressed in MDCK cells. Fold changes in gene expression were calculated using a classical 2^−ΔΔCT^ method. All samples were run in triplicate. The primer sequences used for specific genes are listed in Table 2.

**Table 2:** Primer sequences for quantitative PCR.

Target gene	Direction	Sequence
HA	Forward	5’TTCCCAAGATCCATCCGGCAA 3’
HA	Reverse	5’CCTGCTCGAAGACAGCCACAACG 3’
GAPDH	Forward	5’AGGGCAATGCCAGCCCCAGCG 3’
GAPDH	Reverse	5’AGGCGTCGGAGGGCCCCCTC 3’

### Virus titer reduction assay

Time course of the inhibitory effect of HA-FP-1 against influenza *A/Puerto Rico/8/34* (H1N1) was evaluated by the virus titer reduction assay as previously reported with some modifications [18]. Two different time points for drug administration were utilized in our experiments: (1) the pre-incubation refers that peptides were pre-incubated with virus for 30 min prior to transferring to the MDCK cells; and (2) the post-infection indicates that virus was incubated with MDCK cell for 60 min. Then the cells were washed twice with PBS, and subsequently adding the different concentration of peptides in virus growth medium. At 48 h post-infection, the inhibition of viral replication was measured by determining the virus titer in the supernatant.

To determine the virus titer in the supernatant [19–20], 50 μL supernatants were transferred into to a 96-well black plate followed by addition of 20 μL H_2_O and 30 μL 4-MUNANA substrate (2-(4-methylumbelliferyl)-α-D-N- acetylneuraminic acid sodium) in diluted buffer (33 mM MES (pH = 3.5) and 4 mM CaCl_2_) at a final concentration of 20 μM. The mixtures were incubated at 37°C for 1 h in the dark, then the reaction was terminated with 150 μL 14 mM NaOH (dissolved in 83% ethanol) and the resultant fluorescence was recorded at the excitation wavelength of 340 nm and emission wavelength of 440 nm. The inhibition ratio was obtained using the equation:

$$\text{Inhibitory activity}\;\left(\%\right)=\frac{\left({\text{F}}_{\text{virus}}-{\text{F}}_{\text{sample}}\right)}{\left({\text{F}}_{\text{virus}}-{\text{F}}_{\text{substrate}}\right)}\times 100\%$$

Where F_virus_ is the fluorescence of supernatants of the virus control, F_substrate_ is the fluorescence of the substrate control, and F_sample_ is the fluorescence of the supernatants of the infected cells that were treated with the peptides. IC_50_ was determined by extrapolation of the results from various doses tested using a linear equation.

### Enzyme-linked immunosorbent assay

The inhibitory effect of HA-FP-1 against influenza *A/Puerto Rico/8/34* (H1N1) was further evaluated by enzyme-linked immunosorbent assay (ELISA) [21]. Two different drug administration methods were employed in our experiments, and the procedure is adopted the same as mentioned above: (1) The pre-incubation refers that peptides were pre-incubated with virus for 30 min prior to transferring to the MDCK cells; and (2) The post-infection indicates that virus was incubated with MDCK cells for 60 min. Then the cells were washed twice with PBS, and subsequently adding the virus growth medium (serum free DMEM supplemented with 0.2% BSA, 1 μg/mL TPCK-trypsin). Viral replication was determined by measuring the levels of influenza virus nucleoprotein (NP) using an influenza A virus NCP ELISA kit (Photometric; Virusys Corp.) Percent protection was calculated as [1—(mean OD_450_ compound—mean OD_450_ medium)/(mean OD_450_ DMSO—mean OD_450_ medium)] × 100%, where mean OD_450_ compound, mean OD_450_ medium, and mean OD_450_ DMSO are the absorbance (optical density at 450 nm [OD_450_]) of compound- and virus-containing samples, the absorbance of no-virus control samples, and the absorbance of DMSO- and virus-containing.

### Measurement of the inhibitory activity on the entry of H5N1 pseudovirus

The plasmids encoding HA and NA of *A/Thailand/Kan353/2004* were used to prepare H5N1 pseudovirus. The protocol was adopted as reported previously [22]. In briefly, 293T cells in 6-well plate (60–70% confluent) were co-transfected with 1 μg HA plasmid, 1 μg NA plasmid, and 3 μg HIV backbone plasmid (pNL4-3.luc.R^−^E^−^), which contains an Env and Vpr defective, luciferase-expressing HIV-1 genome per well, using the PEI (polyethylenimine) as a transfection reagent at a ratio of 3:1 to the total weight of the three plasmids. After transfection for 48 h, the culture supernatants were harvested and centrifuged at 2000 rpm for 5 min. Aliquots were stored at -80°C. The amount of pseudotyped particles was quantitated using the luciferase assay.

To measure the inhibitory activities of pFPs, MDCK cells (1×10^4^/well) were seeded in 96-well plates and grown overnight. The peptides were serially two-fold diluted from 100 to 3.12 μg/mL in culture medium and then incubated with equal volume of pseudo-typed particles at 37°C for 30 min. Subsequently, the virus–peptide mixtures were transferred to the cells and incubated for an additional 48 h. Cells were washed with PBS and lysed with the lysing reagent included in the luciferase kit. Aliquots of cell lysates were transferred to 96 well flat bottom luminom, followed by the addition of luciferase substrate. The luciferase activity was measured in a microplate luminometer (Genios Pro Tecan, Swiss). CL-385319 at 50 μM was used as a positive control [23], while wells without peptides as a negative control.

### Measurement of the inhibitory activity on the entry of VSV-G pseudovirus

The VSV pseudovirus was constructed by using VSV-glycoprotein encoded plasmid and HIV backbone plasmid (pNL4-3.luc.R^−^E^−^). The protocol for preparation of pseudovirus and the measurement of the inhibitory activity toward VSV-G pseudovirus were similarly to that of influenza A pseudovirus. MDCK cells (1 × 10^4^/well) were seeded in 96-well plates and incubated overnight. After removal of culture supernatants, 100 μL of fresh cell culture medium was added into the plate. The peptides were serially two-fold diluted from 100 to 0.78 μg/mL in culture medium and then incubated with equal volume of pseudo-typed particles at 37°C for 30 min. Subsequently, the same volume of virus-compound mixture was transferred into the MDCK cells and incubated for 48 h at 37°C before performing luciferase assay as described above.

### Hemagglutination inhibition (HI) assay

The HI assay was employed to evaluate the inhibitory effects of pFPs on viral adsorption into target cells [21]. Twofold serial dilutions of H5 antigen in 25 μL was mixed with equal volume of HA-FP-1 at the final concentration of 50 μg/mL in V-bottomed 96-well micro plates. Subsequently, 50 μL of chicken erythrocytes (0.5% v/v in PBS) were added to each well. An equal volume of HA antiserum (25 μL, 1:20) was used as positive control, while PBS as negative control. The hemagglutination reaction results were read after incubation for 1 h at room temperature. Both H5 standard antigen and antiserum were provided by Harbin Veterinary Research Institute, China.

### Fusion inhibition (FI) assay

The FI assay was carried out to evaluate the antagonistic effects from HA-FP-O to HA-FP-1 on the inhibition of viral entry into target cells. The tests were performed using 25 μg/mL of HA-FP-1, while the concentration of HA-FP-O was varied. The ration of HA-FP-1 and HA-FP-O varied from 1:3, 1:2, 1:1, 1:0.5 and 1:0.3, respectively, and the inhibitory effect on the entry of H5N1 pseudovirus was measured. While the same concentration of peptide HA-FP-1 and HA-FP-O only was used as controls, respectively.

### Hemolysis inhibition assay

The experiment procedure on virus-induced hemolysis at low pH was slightly adopted from a procedure described previously by Luo et al [24]. 100 μL of HA-FP-1 diluted in PBS was mixed with an equal volume of the influenza virus *A/Puerto Rico/8/34* (H1N1) strain (1.5 × 10^8^ TCID_50_/mL) in a 96-deepwell plate and incubated at room temperature for 30 min. Then 200 μL of 2% chicken erythrocytes pre-warmed at 37°C was added, and the mixture was incubated at 37°C for another 30 min. To trigger hemolysis, 100 μL of sodium acetate (0.5 M, pH 5.0) was added and mixed well with the erythrocyte suspension. The mixture was incubated at 37°C for 30 min for HA acidification and hemolysis, and then the plates were centrifuged at 1,200 rpm for 6 min to separate nonlysed erythrocytes. After centrifugation, 300 μL of supernatant was transferred to another flat-bottom 96-well plate. The OD_540_ was recorded on a microtiter plate reader.

### The antiviral activity of the peptides against NA-H274Y mutation virus

MDCK cells were seeded into a 96-well plate at 2 × 10^4^ per well and incubated overnight until grown up to confluent. Influenza strain of *A/Puerto Rico/8/34* (H1N1) with NA-H274Y mutation virus at 100 TCID_50_ were mixed with peptides in 2-fold dilution and incubated at 37°C for 30 min. Subsequently, the virus-peptide mixtures were added to the cells and incubated for another 30 min. Then cells were washed twice with PBS to remove unabsorbed virus, followed by the addition of DMEM supplemented with 1 μg/mL TPCK-trypsin and 0.2% BSA. At 24 h post infection, the cells was immobilized with 80% pre-cooled acetone for 10 min and then the viral replication was determined by measuring the levels of influenza virus nucleoprotein (NP) with ELISA. Percent protection was calculated as [1 - (mean OD_450_ compound—mean OD_450_ medium)/(mean OD_450_ DMSO—mean OD_450_ medium)] × 100%, where mean OD_450_ compound, mean OD_450_ medium, and mean OD_450_ DMSO refer to the absorbance (optical density at 450 nm [OD_450_]) of compound- and virus-containing samples, the absorbance of no-virus control samples, and the absorbance of DMSO- and virus-containing. The IC_50_ values for each peptide were 5.388 ± 3.112 μg/mL (HA-FP-1) and 7.811 ± 1.839 μg/mL (HA-FP-2-1), respectively, while Oseltamivir (Tamiflu) was used as a positive control with IC_50_ value of 0.33 ± 0.037 μg/mL, while for WT of influenza strain of *A/Puerto Rico/8/34* (H1N1), the IC_50_ value is 0.017 ± 0.002 μg/mL.

### Statistical analysis

Graph Pad Prism 5 (San Diego, CA) was used to determine the half cytotoxic concentration (CC_50_) and half inhibitory concentration (IC_50_) values of peptides. All IC_50_ and CC_50_ values were calculated as the means ± standard error of the mean (SEM) from triplicate assay from at least three independent experiments. The data was determined by one-way ANOVA method by using SPSS software. Statistical significance was defined as *p* < 0.05 (*p < 0.01, **p < 0.001).

## Results

### Positively charged “pseudo” FPs (pFPs) inhibit the replication of influenza A viruses

Peptides were designed from the fusogenic peptides (FPs) of influenza A viruses by replacing negatively or neutrally charged residues with lysines, meanwhile, kept most other residues intact, as indicated in Table 3. Given the highly conserved region of FPs in the HAs (Table 1) and the critical role in fusogenic process [7], we then evaluated their antiviral activity against influenza viral strains of *A/Puerto Rico/8/34* (H1N1) and *A/Aichi/2/68* (H3N2) by using virus cytopathic effect (CPE) inhibition assay. As shown in Table 3, when the virus was exposed to peptides for 30 min prior to infection of MDCK cells, the antiviral activities of HA-FP-1 toward tested viral strains were observed to be 9.61±1.15 (H1N1) and 5.90±0.62 (H3N2) μg/mL, respectively.

**Table 3:** Peptides derived from influenza type A viruses and their antiviral activities.

Name	Sequence^a^	Subtype	Charge	H1N1^b^	H3N2^c^	CC_50_ (μg/mL)	CC_50_ (μg/mL)
				IC_50_ (μg/mL)	IC_50_ (μg/mL)	without serum	with serum
HA-FP-O	GLFGAIAGFI**E**NGW**E**GMI **D**G	H3	-3	NA^d^	NA	NT^d^	NT
HA-FP-1	GLFGAIAGFI**K**NGW**K**GMI **K**G	H3	+3	9.61±1.15	5.90±0.62	111.70±0.40	143.65±0.63
HA-FP-2	GLFGAIAGFI**K**GGW**Q**GMV**K**G	H5	+2	11.96±0.80	15.64±0.11	NT	NT
HA-FP-2-1	GLFGAIAGFI**KK**GW**K**GMV**K**G	H5	+4	11.19±0.63	28.17±1.96	162.0±1.13	198.46±0.55
HA-FP-3	GLFGAIAGFI**K**GGWP GL V**K**G	H9	+2	15.17±3.69	34.91±4.21	NT	NT
HA-FP-3-1	GLFGAIAGFI**KK**GWP GL V**K**G	H9	+3	10.37±0.05	27.57±0.43	174.53±1.90	209.35±0.21

^a^ All C-termini were amidated, and the molecular weight calculation was based on: http://www.peptidesynthetics.co.uk/tools/

^b^ *A/Puerto Rico/8/34*(H1N1). Antiviral effects were determined by viral cytopathic effect (CPE) inhibition assay and Ribavirin was used as a positive control (IC_50_: 3.22±0.76 μg/mL).

^c^ *A/Aichi/2/68* (H3N2). Antiviral results were obtained by CPE inhibition assay and Ribavirin as a positive control (IC_50_: 0.63±0.018 μg/mL).

^d^ NA: not active; NT: not test.

**Fig 1:**
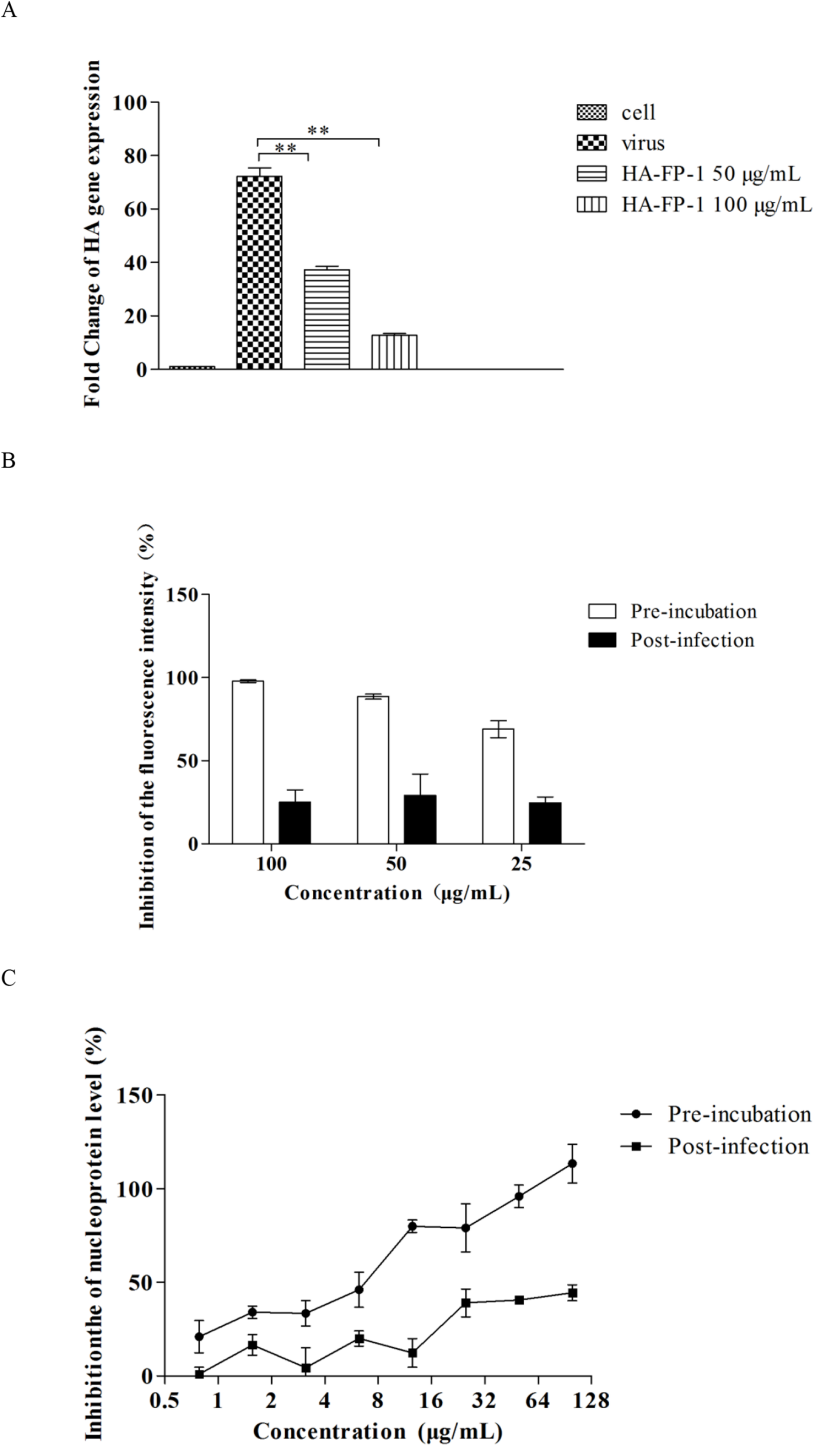
The antiviral effects of HA-FP-1. (A) Viral HA replication was detected by quantitative real-time PCR. The influenza *A/Puerto Rico/8/34* (H1N1) virus at 100 TCID_50_ was incubated with peptides for 30 min prior to infection. At 24 h post infection, the total RNA was reverse transcribed into cDNA and performed real time PCR. (B) Antiviral effects of HA-FP-1 against influenza *A/Puerto Rico/8/34* (H1N1) by virus titer reduction assay. The pre-incubation (white bars) refers that peptides were pre-incubated with virus for 30 min prior to transferring to the MDCK cells. While the post-infection (black bars) indicates that virus was incubated with MDCK cell for 30 min, then the cells were washed twice with PBS, and subsequently adding the virus growth medium containing various concentrations of peptides. At 48 h post infection, the inhibition of viral replication was evaluated by measuring the enzymatic activity of neuraminidase. (C) Antiviral effects of HA-FP-1 against influenza *A/Puerto Rico/8/34* (H1N1). The virus were tested with peptides by pre-incubated and post-infection, at 48 h treatment, the inhibition of viral replication was evaluated by enzyme-linked immunosorbent assay.

In parallel to the measurement of CPE inhibition test, the antiviral effect of HA-FP-1 against influenza *A/Puerto Rico/8/34* (H1N1) was further assessed by employing quantitative real time PCR (Fig 1A). As influenza hemagglutinin protein is one of the essential viral proteins directly associated with virus replication, we then inspected the inhibition on mRNA levels of HA gene. As a result, a reduced level of mRNA expression upon treatment with HA-FP-1 was observed, consistent with the results from CPE inhibition assay.

On the basis of this progress, we were wondering how the antiviral effect of pFPs would be if tested with drug-resistant viral strain of *A/Puerto Rico/8/34* (H1N1) with NA-H274Y mutation, an influenza A (H1N1) virus with NA-H274Y neuraminidase mutation which is known for its resistance to neuraminidase inhibitor of Oseltamivir [25].

After treatment of virus with peptides similar to that in CPE inhibition assay, the antiviral effect was determined by measuring the levels of influenza virus nucleoprotein (NP) with enzyme-linked immunosorbent assay (ELISA). As a consequence, the IC_50_ value of HA-FP-1 against NA-H274Y mutation virus was 5.388 ± 3.112 μg/mL, similar to the results obtained from other assays.

### HA-FP-1 inhibits the replication of virus in the early stage of infection

To confirm the antiviral activities from CPE test and RT-PCR measurement, we then evaluated the antiviral effect of HA-FP-1 toward *A/Puerto Rico/8/34* (H1N1) viral strain by using virus titer reduction assay (Fig 1B) and ELISA (Fig 1C). In addition, to get more details regarding the possible mechanism by which to inhibit the replication of influenza A virus, we employed two different time points for drug administration in these experiments: the pre-incubation and the post-infection. Consequently, the virus inhibitory effects in both Fig 1B and 1C were clearly observed with a dose-dependent manner. In particular, the antiviral activities from pre-incubation were much more potent than from post infection, implying the antiviral activity may result from the inhibition of the early stage of virus infection.

### pFPs inhibit the entry of H5N1 influenza A pseudovirus

The initial results inspired us to further investigate the detailed mechanism by which to inhibit the replication of influenza virus. Herein, we employed H5N1 pseudovirus as our “entry inhibitor” model to evaluate the antiviral effects of these peptides. The pseudovirus was constructed by using the plasmids encoding HA and NA of *A/Thailand/Kan353/2004*, by which the antiviral effect was evaluated by measuring the inhibitory effect on the infection of H5N1 pseudovirus [23]. Assays were carried out with virus in the presence of vehicle (H_2_O), or in the presence of various concentrations of peptides.

**Fig 2:**
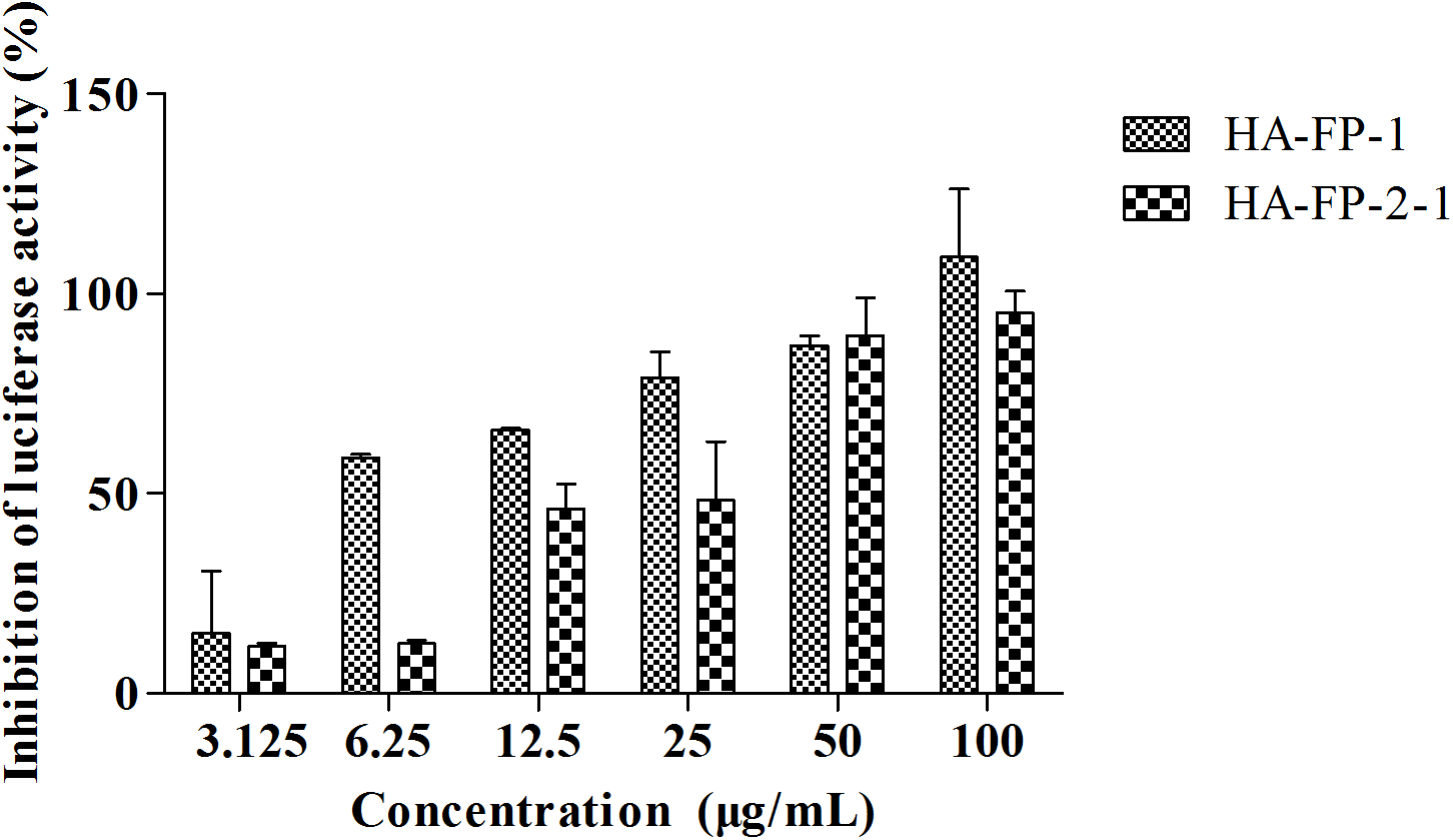
Inhibitory effect of pFPs against influenza A pseudovirus. The plasmids encoding HA and NA of *A/Thailand/Kan353/2004* were used to prepare H5N1 pseudoviruses. To measure the inhibitory effects of tested peptides, MDCK cells (1 × 10^4^/well) were seeded in 96-well plates and grown overnight. The peptides in various concentrations were incubated with pseudo-typed particles for 30 min at 37°C, then transferred to the MDCK cells and incubated for an additional 48 h. The luciferase activity expressed by pseudo-virus was measured. CL-385319 at 50 μM was used as a positive control, wells without peptides as a negative control.

As shown in Fig 2, HA-FP-1 and HA-FP-2-1 exhibited the highest inhibitory effects on the infection of H5N1 influenza A pseudovirus on MDCK cells with IC_50_ of 7.05 and 18.09 μg/mL respectively, while others including the prototype of HA-FP-O only showed a lower or no inhibition in our test range. Obviously, these data indicated a promising approach in construction of antiviral agents by which to block the entry of pathogenic viruses into host cells.

### pFPs are unable to inhibit the entry of vesicular stomatitis (VSV) pseudovirus

Although the pFPs exhibited an inhibitory effect toward pseudo-influenza A virus, we were not sure how the specificity they were and what the drug target might be. In this study, the pseudo-influenza A virus was constructed by encoding HA and NA plasmids of IAV into HIV backbone. Therefore, the inhibitory effects may also result from the interaction of peptide with HIV backbone. Given the fact that both VSV-G and IAV take the same route of endocytosis for cellular entry, we then employed VSV-G as a negative control to study the specificity of pFPs. The vesicular stomatitis pseudovirus was constructed by using VSV-glycoprotein encoded plasmid similar to that of IAV pseudovirus, and then was used to screen pFPs for the inhibitory effect.

**Fig 3:**
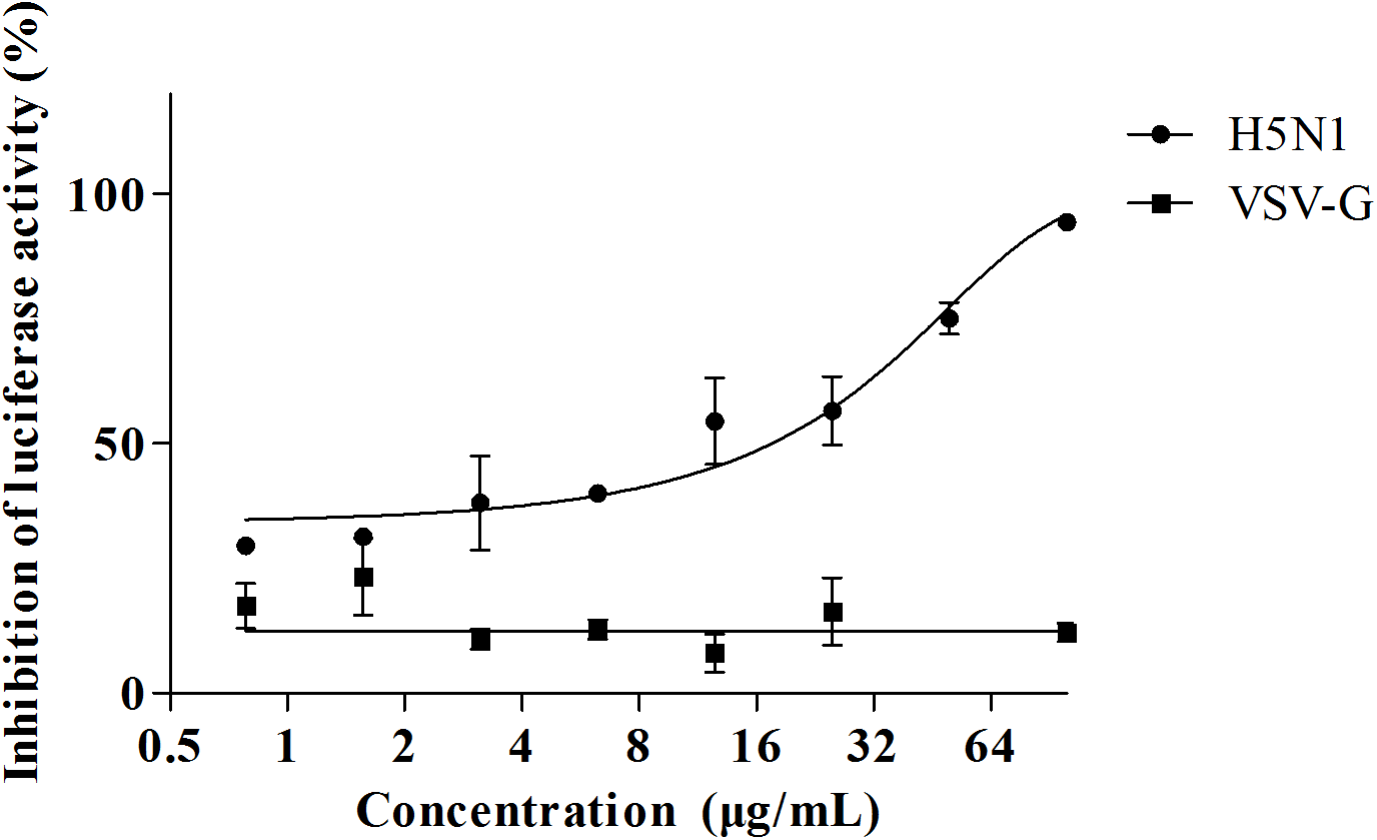
The inhibitory effect of HA-FP-1 toward VSV-G and H5N1 pseudoviruses was compared at various concentrations. MDCK cells (1 × 10^4^/well) were seeded in 96-well plates and incubated overnight. After removal of culture supernatants, 100 μL of fresh cell culture medium was added into the plate. The peptides were serially two-fold diluted from 100 to 0.78 μg/mL in culture medium and then incubated with equal volume of pseudo-typed particles at 37°C for 30 min. Subsequently, the same volume of virus-peptide mixture was transferred into the MDCK cells and incubated for 48 h at 37°C before performing luciferase assay.

The experiment data showed that none of these peptides was able to reduce the infectivity of VSV-G pseudovirus on MDCK cells even at the highest concentration tested (100 μg/mL) (Fig 3). Hence, these data provide evidences that pFPs may selectively inhibit the entry of influenza A viruses.

### The target of pFPs is not sialylglycoconjugate receptors of HA

**Fig 4:**
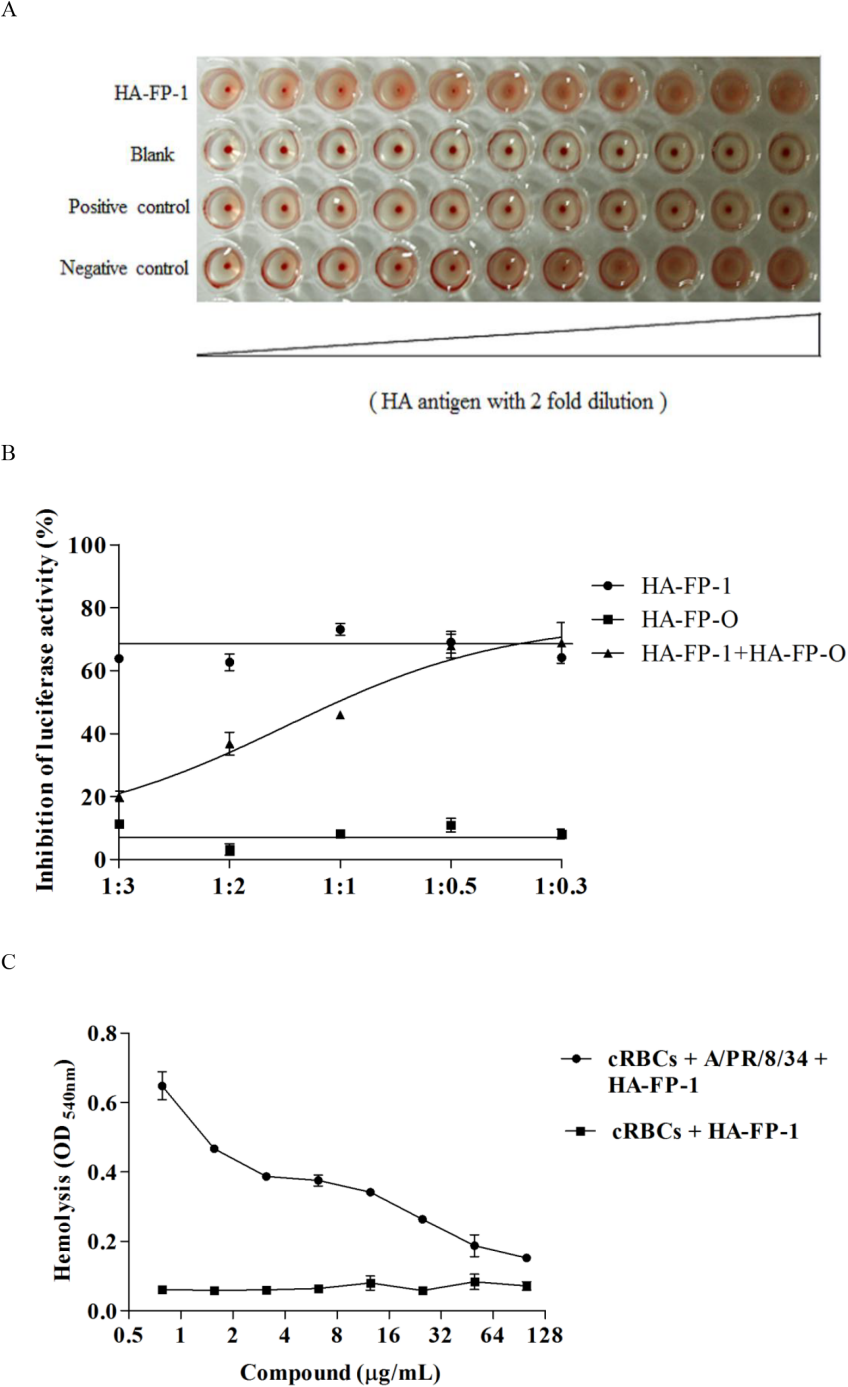
The interaction of the fusion peptide with influenza hemagglutinin. (A) HI assay was employed to evaluate the inhibitory effects of pFPs on viral adsorption into target cells. Twofold serial dilutions of H5 antigen in 25 μL was mixed with equal volume of HA-FP-1 at the final concentration of 50 μg/mL in V-bottomed 96-well micro-plates. Subsequently, 50 μL of chicken erythrocytes (0.5% v/v in PBS) were added to each well. HA antiserum (25 μL, 1:20) was used as positive control, while PBS as negative control. (B) Fusion inhibition assay. The inhibitory effect on the entry of H5N1 pseudovirus was measured by using the mixture of HA-FP-1 and HA-FP-O in various ratios. The concentration for HA-FP-1 was 25 μg/mL., while for HA-FP-O was varied. The ration between HA-FP-1 and HA-FP-O was 1:3, 1:2, 1:1, 1:0.5 and 1:0.3, respectively. (C) Hemolysis inhibition assay. Two-fold serially diluted peptide HA-FP-1 in PBS was mixed with an equal volume of the influenza virus *A/Puerto Rico/8/34* (H1N1) strain (1.5 × 10^8^ TCID_50_/mL) in a 96-well plate. 200 μL of 2% chicken erythrocytes pre-warmed at 37°C was then added. The mixture was incubated at 37°C for another 30 min. To trigger hemolysis, 100 μL of sodium acetate (0.5 M; pH 5.0) was added and mixed well with the erythrocyte suspension. The mixture was incubated at 37°C for 30 min for HA acidification and hemolysis. To separate nonlysed erythrocytes, plates were centrifuged at the end of incubation at 1,200 rpm for 6 min.

Thus far, sialic acid (N-acetyl neuraminic acid) is the only known essential component of cellular receptors for influenza type A virus [26]. To determine whether our pFPs were binding to the sialic acid-binding site on HA, we then carried out an HA inhibition assay (HI assay). As shown in Fig 4A, twofold serial dilutions of H5 antigen in 25 μL was mixed with equal volume of HA-FP-1 in V-bottomed 96-well micro-plates. Subsequently, 50 μL of chicken erythrocytes (0.5% v/v in PBS) were added to each well. As a consequence, HA-FP-1 was unable to inhibit the agglutination of red blood cells, indicating the binding site of these peptides would not be the sialic acid binding site on HA1 subunit of HA [27].

### Peptide HA-FP-O antagonizes the antiviral activity of HA-FP-1

The hemagglutinin (HA) is comprised of two subunits, HA1 and HA2. Since the HI assay has excluded the possible target of sialic acid-binding site on HA1, we next investigated whether the N-terminal region of HA2 was the possible target of these peptides. The fusion inhibition assay (FI assay) was employed by measuring the competition effect exerted from HA-FP-O to HA-FP-1 (Fig 4B). The concentration of HA-FP-1 was 25 μg/mL, while HA-FP-O was varied from 75 to 6.25 μg/mL. The results showed that as the concentration ration between HA-FP-1 and HA-FP-O reached 1:3 the antiviral activity of HA-FP-1 was completely abolished (Fig 4B), indicating the N-terminal region of HA2 as the possible target of pFPs.

### HA-FP-1 blocks the lysis of erythrocytes induced by acidic condition

Membrane fusion induced by low pH is a key step in the infection process of IAV. To investigate whether pFPs inhibit the viral fusion step by blocking the conformational changes of HA2, we then carried out a hemolysis inhibition assay under acidic condition. The data showed that when exposure the mixture of chicken red blood cells (cRBCs) with influenza virus strain of *A/Puerto Rico/8/34* (H1N1) (1.5 × 10^8^ TCID_50_/mL) to the two-fold serial dilutions of HA-FP-1 in acidic buffer (pH = 5), the hemolysis of cRBCs was apparently inhibited in a dose-dependent manner, suggesting that interruption of the conformational changes of HA2 induced by low pH would be the possible mechanism of HA-FP-1, by which to block the entry of virus (Fig 4C).

## Discussion

With the appearance of drug resistant influenza A viral strains, it is imperative to develop new antiviral drugs or strategies effectively targeting these seasonal pathogens. In this regard, we focus our interest on the development of antiviral agents from antimicrobial peptides. The feasibility of this strategy has been well documented by a number of literatures [18, 28, 29], which proposed that the general antiviral mechanisms may involve the direct virolysis, inhibition of transcription from the long terminal repeat (LTR) promoter, and block of cell entry by binding to cell surface receptors [4]. With respect to the cell “entry blocker”, the clinically used HIV entry inhibitor of enfuvirtide (Fuzeon) has shown a great potential of peptide drug in the prevention of viral infections [5].

Spurred by this success, in this paper, we demonstrate several effective “virus entry inhibitors” derived from fusogenic peptides of hemagglutinin glycoprotein of influenza A viruses. The fusion peptides were reported to involve the disruption and destabilization of cellular membrane similar to those of conventional antimicrobial peptides. Thus, we turned them into potent antimicrobials by reversing the charges of these peptides. Interestingly, not only did they show potent bactericidal activities [11], but these positively charged fusion peptides also inhibited the infection of influenza A viruses, whereas were unable to block the entry of VSV-G to host cells, indicating multiple functions in their antimicrobial activities.

In our previous work [11], we have reported the cytotoxicity of these peptides toward MDCK cells, as well as toward fresh rabbit red blood cells (rRBCs). The results showed that the CC_50_ values against MDCK of HA-FP-1 and HA-FP-2-1 were 144.4 μg/mL (HA-FP-1) and 195.1 μg/mL (HA-FP-2-1), respectively, while the hemolytic activity against rRBCs was lower than 10% at 125 μg/mL. Therefore, considering the micro-molar range of antiviral activity, the therapeutic indices were not very high. However, as a proof-of-concept and preliminary data, these peptides clearly showed the inhibitory effect in preventing the infection from IAV, as well as the potentials in development as antiviral agents. Currently, in order to improve their antiviral efficacy, the structural modifications are under investigation by applying several strategies, such as extending the peptide sequences to 23 amino acids or longer, adding a hydrophobic molecule or residues to the N- and C-termini respectively. With these efforts, we anticipated that the more potent antiviral peptides would be obtained and the higher therapeutic indices would be achieved.

**Fig 5:**
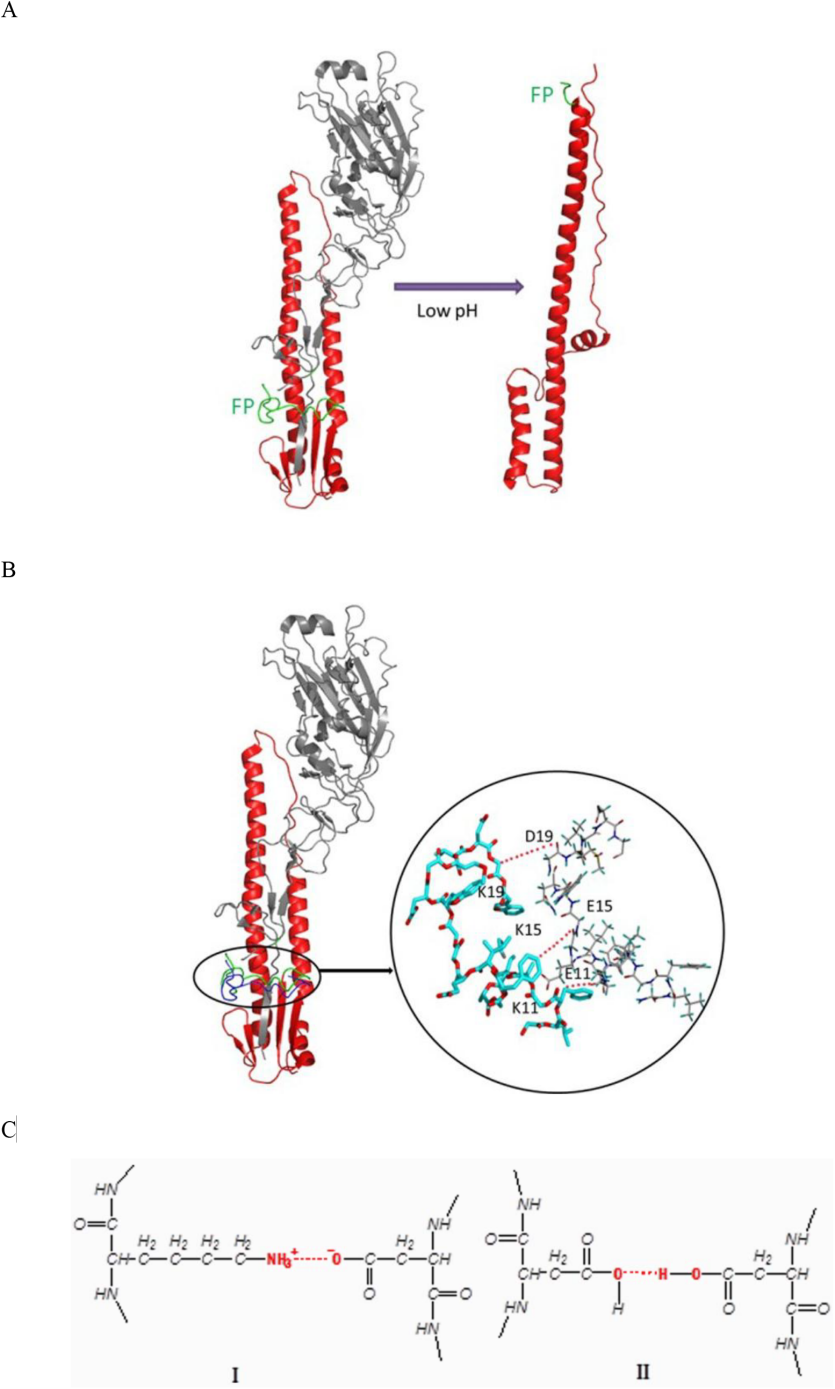
Hypothesized molecular interactions between pFPs and HA2. (A) The membrane fusion process of IAV is initiated via the conformational changes of HA induced under the acidic condition (green color refers to fusogenic domain of HA2). (B) With the insertion of an exogenous segment of HA-FP-1 (blue color) into the N-terminal region of fusogenic domain of HA2 (green color), the conformational changes were blocked, and the fusion process was interrupted. (C) The interactions between HA-FP-1 and fusogenic domain of HA2 include salt bridges and hydrogen bonds (I), while only hydrogen bond interactions were observed between HA-FP-O and fusogenic domain of HA2 (II).

The mechanism study indicated that the antiviral activity was resulted from the interactions of pFPs with HA by inhibition of the entry of virus. However, the detailed study showed that the binding site of these peptides was not the HA1 subunit, a well known binding site responsible for sialic acid receptor on the host cells’ membrane [30]. Instead, these pFPs may interact with HA2 subunit of HA as deduced from the fusion inhibition and hemolysis inhibition assays. Due to the critical role of fusion peptides (FPs) in the process of virus entry, it is reasonably proposed that the different inhibitory effects of pFPs toward IAV would be associated with the different affinities between the pFPs and hemagglutinin glycoproteins (HAs) of IAV. As a result, the stronger of the affinity is, the higher of the inhibitory effect might be. Based on these results, therefore, we deduced that the possible interactions between HA and pFPs might be the N-terminal region of HA2 as described in Fig 5.

Generally, the membrane fusion process of IAV is initiated *via* the conformational changes of HA2 subunit induced by the acidic condition (pH = 5) (Fig 5A) [28, 31]. However, with the insertion of an exogenous segment of positively charged pFP into the N-terminal region of fusogenic domain of HA2, the conformational changes were blocked, subsequently the fusion process was interrupted, and the viral entry was terminated (Fig 5B). Hence, the antiviral effect was subject to the affinities between the exogenous pFPs and HA2 subunit, of which, the positively charged lysinyl residues at position 11, 15 and 19 of HA-FP-1 exerted strong interactions with the negatively charged residues of glutamic acid/aspartic acid from the same positions of HA2 (Fig 5C-I) *via* both salt bridges and hydrogen bonds. In contrast, the predominant interactions between negatively charged HA-FP-O and HA2 would be hydrogen bonds (Fig 5C-II), which conveyed a much weaker interactions than the former. Consequently, the strong interactions between HA-FP-1 and HA2 would “freeze” the conformational changes of HA2, and subsequently inhibit the entry of IAV.

Taken together, in the current study, we provide evidences that positively charged pFPs are effective viral entry blockers besides their potent antimicrobial activities. In addition, this paper also describes a promising “complementary” strategy in designing viral “entry blockers” by rationally tuning some residues derived from a conserved region of viral envelop protein, such as from hemagglutinin glycoprotein of IAV or gp160 of HIV. This approach involving the formation of acid-base pairs might be able to enhance the interactions between the viral proteins and antiviral agents thereby blocking the entry of virus, which might be useful in our current war against those urgent pathogenic microorganisms such as IAV and HIV. In particular, these multi-functional antimicrobial agents would be useful in treatment of bacterial co-infection during pandemic influenza periods.

## References

[1] Nguyen T, Rivailler P, Davis CT, Hoa do T, Balish A, Dang NH, et al Evolution of highly pathogenic avian influenza (H5N1) virus populations in Vietnam between 2007 and 2010. Virology. 2012;432(2):405–16. 10.1016/j.virol.2012.06.021 22818871

[2] Guan Y, Farooqui A, Zhu H, Dong W, Wang J, Kelvin DJ. H7N9 Incident, immune status, the elderly and a warning of an influenza pandemic. Journal of infection in developing countries. 2013;7(4):302–7. 10.3855/jidc.3675 23592638

[3] Aoki W, Kuroda K, Ueda M. Next generation of antimicrobial peptides as molecular targeted medicines. Journal of bioscience and bioengineering. 2012;114(4):365–70 10.1016/j.jbiosc.2012.05.001 22658802

[4] Gratton JP, Yu J, Griffith JW, Babbitt RW, Scotland RS, Hickey R, et al Cell-permeable peptides improve cellular uptake and therapeutic gene delivery of replication-deficient viruses in cells and in vivo. Nature medicine. 2003;9(3):357–62. 12598894 10.1038/nm835

[5] Liu S, Lu H, Niu J, Xu Y, Wu S, Jiang S. Different from the HIV fusion inhibitor C34, the anti-HIV drug Fuzeon (T-20) inhibits HIV-1 entry by targeting multiple sites in gp41 and gp120. The Journal of biological chemistry. 2005;280(12):11259–73. 15640162 10.1074/jbc.M411141200

[6] Harrison SC. Viral membrane fusion. Virology. 2015; 479–480: 498–507. 10.1016/j.virol.2015.03.043 25866377 PMC4424100

[7] Huang Q, Korte T, Rachakonda PS, Knapp EW, Herrmann A. Energetics of the loop-to-helix transition leading to the coiled-coil structure of influenza virus hemagglutinin HA2 subunits. Proteins. 2009;74(2):291–303. 10.1002/prot.22157 18618705

[8] Nobusawa E, Aoyama T, Kato H, Suzuki Y, Tateno Y, Nakajima K. Comparison of complete amino acid sequences and receptor-binding properties among 13 serotypes of hemagglutinins of influenza A viruses. Virology. 1991;182(2):475–85. 2024485 10.1016/0042-6822(91)90588-3

[9] Siegel DP, Epand RM. Effect of influenza hemagglutinin fusion peptide on lamellar/inverted phase transitions in dipalmitoleoylphosphatidylethanolamine: implications for membrane fusion mechanisms. Biochimica et biophysica acta. 2000;1468(1–2):87–98. 11018654 10.1016/s0005-2736(00)00246-7

[10] Ilic N, Novkovic M, Guida F, Xhindoli D, Benincasa M, Tossi A, et al Selective antimicrobial activity and mode of action of adepantins, glycine-rich peptide antibiotics based on anuran antimicrobial peptide sequences. Biochimica et biophysica acta. 2013;1828(3):1004–12. 10.1016/j.bbamem.2012.11.017 23196344

[11] Wang J, Zhong W, Lin D, Xia F, Wu W, Zhang H, et al Antimicrobial Peptides Derived from Fusion Peptides of Influenza A Viruses, a Promising Approach to Designing Poten Antimicrobial Agents. Chemical biology & drug design. 2015; 10.1111/cbdd.12511 25581878

[12] Vaccaro L, Cross KJ, Kleinjung J, Straus SK, Thomas DJ, et al Plasticity of Influenza Haemagglutinin Fusion Peptides and Their Interaction with Lipid Bilayers. Biophysical Journal. 2005; 88: 25–36. 15475582 10.1529/biophysj.104.044537 PMC1305003

[13] White JM, Delos SE, Brecher M, Schornberg K. Structures and mechanisms of viral membrane fusion proteins: multiple variations on a common theme. Critical reviews in biochemistry and molecular biology. 2008;43(3):189–219. 10.1080/10409230802058320 18568847 PMC2649671

[14] Reed L, Muench H. A simple method of estimating fifty percent endpoint. Am J Hyg. 1938;27:493–7.

[15] Fang Y, Zhong W, Wang Y, Xun T, Lin D, Liu W, et al Tuning the antimicrobial pharmacophore to enable discovery of short lipopeptides with multiple modes of action. European journal of medicinal chemistry. 2014;83:36–44. 10.1016/j.ejmech.2014.06.003 24946217

[16] Zhu L, Li Y, Li S, Li H, Qiu Z, Lee C, et al Inhibition of influenza A virus (H1N1) fusion by benzenesulfonamide derivatives targeting viral hemagglutinin. PloS one. 2011;6(12):e29120 10.1371/journal.pone.0029120 22195002 PMC3240648

[17] Khare D, Godbole NM, Pawar SD, Mohan V, Pandey G, Gupta S, et al Calcitriol [1, 25[OH]2 D3] pre- and post-treatment suppresses inflammatory response to influenza A (H1N1) infection in human lung A549 epithelial cells. European journal of nutrition. 2013;52(4):1405–15. 10.1007/s00394-012-0449-7 23015061

[18] Matsubara T, Onishi A, Saito T, Shimada A, Inoue H, Taki T, et al Sialic acid-mimic peptides as hemagglutinin inhibitors for anti-influenza therapy. Journal of medicinal chemistry. 2010;53(11):4441–9. 10.1021/jm1002183 20476787

[19] Liu AL, Wang HD, Lee SM, Wang YT, Du GH. Structure-activity relationship of flavonoids as influenza virus neuraminidase inhibitors and their in vitro anti-viral activities. Bioorganic & medicinal chemistry. 2008;16(15):7141–7. 18640042 10.1016/j.bmc.2008.06.049

[20] Potier M, Mameli L, Belisle M, Dallaire L, Melancon SB. Fluorometric assay of neuraminidase with a sodium (4-methylumbelliferyl-alpha-D-N-acetylneuraminate) substrate. Analytical biochemistry. 1979;94(2):287–96. 464297 10.1016/0003-2697(79)90362-2

[21] Basu A, Antanasijevic A, Wang M, Li B, Mills DM, Ames JA, et al New small molecule entry inhibitors targeting hemagglutinin-mediated influenza a virus fusion. Journal of virology. 2014;88(3):1447–60. 10.1128/JVI.01225-13 24198411 PMC3911584

[22] Zhu Z, Li R, Xiao G, Chen Z, Yang J, Zhu Q, et al Design, synthesis and structure-activity relationship of novel inhibitors against H5N1 hemagglutinin-mediated membrane fusion. European journal of medicinal chemistry. 2012;57:211–6. 10.1016/j.ejmech.2012.08.041 23059548

[23] Liu S, Li R, Zhang R, Chan CC, Xi B, Zhu Z, et al CL-385319 inhibits H5N1 avian influenza A virus infection by blocking viral entry. European journal of pharmacology. 2011;660(2–3):460–7. 10.1016/j.ejphar.2011.04.013 21536025

[24] Lee KK, Pessi A, Gui L, Santoprete A, Talekar A, et al Capturing a fusion intermediate of influenza hemagglutinin with a cholesterol-conjugated peptide, a new antiviral strategy for influenza virus. J Biol Chem. 2011; 286: 42141–42149. 10.1074/jbc.M111.254243 21994935 PMC3234914

[25] Kawai N, Ikematsu H, Iwaki N, Kondou K, Hirotsu N, Kawashima T, et al Clinical effectiveness of oseltamivir for influenza A(H1N1) virus with H274Y neuraminidase mutation. The Journal of infection. 2009;59(3):207–12. 10.1016/j.jinf.2009.07.002 19619898

[26] Yang J, Li M, Shen X, Liu S. Influenza A virus entry inhibitors targeting the hemagglutinin. Viruses. 2013;5(1):352–73. 10.3390/v5010352 23340380 PMC3564125

[27] Gunther-Ausborn S, Schoen P, Bartoldus I, Wilschut J, Stegmann T. Role of hemagglutinin surface density in the initial stages of influenza virus fusion: lack of evidence for cooperativity. Journal of virology. 2000; 74: 2714–2720. 10684287 10.1128/jvi.74.6.2714-2720.2000 PMC111761

[28] Francis JN, Bunce CJ, Horlock C, Watson JM, Warrington SJ, Georges B, et al A novel peptide-based pan-influenza A vaccine: a double blind, randomised clinical trial of immunogenicity and safety. Vaccine. 2015;33(2):396–402. 10.1016/j.vaccine.2014.06.006 24928790

[29] Hoffmann J, Schneider C, Heinbockel L, Brandenburg K, Reimer R, Gabriel G. A new class of synthetic anti-lipopolysaccharide peptides inhibits influenza A virus replication by blocking cellular attachment. Antiviral research. 2014;104:23–33. 10.1016/j.antiviral.2014.01.015 24486207

[30] Mair CM, Meyer T, Schneider K, Huang Q, Veit M, Herrmann A. A histidine residue of the influenza virus hemagglutinin controls the pH dependence of the conformational change mediating membrane fusion. Journal of virology. 2014;88(22):13189–200. 10.1128/JVI.01704-14 25187542 PMC4249083

[31] Li R, Song D, Zhu Z, Xu H, Liu S. An induced pocket for the binding of potent fusion inhibitor CL-385319 with H5N1 influenza virus hemagglutinin. PloS one. 2012;7(8):e41956 10.1371/journal.pone.0041956 22876294 PMC3410875

